# Advantages of laparoscopic segmentectomy of the liver using ICG fluorescent navigation by the negative staining method: A comparison with open procedure

**DOI:** 10.1002/ags3.12786

**Published:** 2024-03-07

**Authors:** Kenichiro Araki, Akira Watanabe, Norifumi Harimoto, Takamichi Igrashi, Mariko Tsukagoshi, Norihiro Ishii, Shunsuke Kawai, Kei Hagiwara, Kouki Hoshino, Ken Shirabe

**Affiliations:** ^1^ Division of Hepatobiliary and Pancreatic Surgery, Department of General Surgical Science Gunma University Graduate School of Medicine Maebashi Gunma Japan

**Keywords:** ICG fluorescent imaging, laparoscopic liver resection, laparoscopic segmentectomy of the liver, negative staining method, posterosuperior segment

## Abstract

**Aim:**

Laparoscopic segmentectomy (LS) using indocyanine green (ICG) fluorescence navigation with negative staining method has potential for performing accurate and safe anatomical excision. This study aimed to evaluate the significance of LS using ICG fluorescence navigation compared with open segmentectomy (OS).

**Methods:**

Eighty‐seven patients who underwent anatomical segmentectomies were evaluated for OS (*n* = 44) and LS (*n* = 43). The Glissonean pedicle approach was performed using either extra‐ or intrahepatic method, depending on the location of segment in LS. After clamping pedicle, negative staining method was performed. Liver transection was done along intersegmental plane visualizing by overlay mode of ICG camera. Surgical outcomes were compared between two groups. Correlation between predicted resecting liver volume (PRLV) calculated using volumetry and actual resected liver volume (ARLV) was assessed in two groups.

**Results:**

Patients who underwent LS showed better outcomes in operative time, blood loss, and length of hospital stay. There were significantly fewer Grade II and Grade III or higher postoperative complications in LS group. Both values of AST (*p* < 0.001) and ALT (*p* < 0.001) on postoperative day 1 were significantly lower in LS group than in OS group. PRLV and ARLV were more strongly correlated in LS (*r* = 0.896) than in OS (*r* = 0.773). The difference between PRLV and ARLV was significantly lower in LS group than in OS group (*p* = 0.022), and this trend was particularly noticeable in posterosuperior segment (*p* = 0.008) than in anterolateral segment (*p* = 0.811).

**Conclusion:**

LS using ICG navigation allows precise resection and may contribute to safer short‐term outcomes than OS, particularly in posterosuperior segment.

## Graphical abstract

This study aimed to evaluate the significance of laparoscopic segmentectomy (LS) of the liver using ICG fluorescence navigation compared with open segmentectomy (OS). LS using ICG navigation allows precise resection and may contribute to safer short‐term outcomes than conventional OS, particularly in the posterosuperior segment.

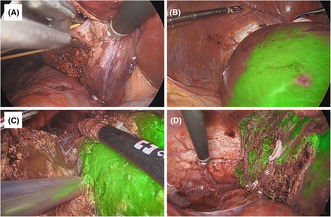

## 1 INTRODUCTION

Laparoscopic liver resection (LLR) has become widespread after two international consensus meetings and is now performed for anatomical resection and major hepatectomies.^1^, ^2^ Anatomical resection has been an oncologically effective surgical procedure for hepatocellular carcinoma since the era of open liver resection,^3^ and is sometimes performed for colorectal liver metastases due to a large tumor diameter or bile duct invasion.^4^ Under such circumstances, anatomical segmentectomy of the liver, which can spare the liver parenchyma compared to major hepatectomy, has long been advocated and performed as a useful surgical procedure for liver tumors associated with deterioration of the liver functional reserve.^5^, ^6^ Since it was first reported that laparoscopic segmentectomy (LS) of a single segment was technically performed in all segments by Ishizawa et al.,^7^ LS has been performed at high‐volume centers, even for highly difficult procedures such as posterosuperior (PS) segments S7 and S8.^8^

In open liver resection, anatomical segmentectomy is mainly performed with reference to the demarcation line owing to ischemic changes on the liver surface, which are visualized by clamping or dividing the Glissonean pedicles. With the advent of ICG fluorescence and the spread of LLR, this concept is undergoing a revolutionary change.^9^, ^10^, ^11^ In LLR, using a new‐generation infrared fluorescence (IR) camera developed through technological innovation, this surgery can be performed with real‐time navigation while sharing ICG fluorescence images on the same surgical screen using a laparoscope.^12^ That is, the intersegmental plane was shared with all participating surgeons, and they can perform liver transection while viewing this image. If LS enables a more accurate resection in the portal territories than conventional methods, it may contribute to postoperative safety. In this study, we evaluated the usefulness of LS using ICG fluorescent navigation and compared it with an open procedure.

## 2 MATERIALS AND METHODS

### 2.1 Patient selection and study protocol

This study included 673 patients who underwent liver resection at our hospital between January 2016 and March 2023. Among the patients who underwent liver resection, we excluded those who underwent combined resection of other organs, bile duct reconstruction, and hepatopancreatoduodenectomy (HPD) cases. Eighty‐seven patients who underwent segmentectomy for Couinaud single (mono) segment were included in this study. Forty‐four patients underwent open segmentectomy (OS), and 43 underwent LS. Patient characteristics and surgical procedures are shown in Table 1. We evaluated the following variables: age; sex; body mass index (BMI); body surface area (BSA); and laboratory findings, including liver function, preoperative diagnosis, and location of the resected segment. We compared the characteristics, surgical outcomes, and accuracy of the predicted resecting liver volume (PRLV) between the OS and LS groups by calculating the preoperative 3D volume and validating the actual resected liver volume of the liver specimen. We also compared the results between the OS and LS groups in patients with the PS segment.

**TABLE 1:** Patient characteristics between OS and LS using ICG navigation.

	OS (*n* = 44)	LS using ICG navigation (*n* = 43)	*p* Value
Variables			
Age, years	69 ± 10	70 ± 9	0.460
Sex male	36 (81.8)	33 (76.7)	0.559
BMI	22.9 ± 3.5	24.0 ± 3.2	0.316
BSA, m^2^	1.60 ± 0.24	1.57 ± 0.17	1.000
Diagnosis			
Hepatocellular carcinoma	24 (54.5)	19 (44.2)	0.334
Colorectal liver metastases	11 (25.0)	16 (37.2)	0.218
Intrahepatic cholangiocarcinoma	2 (4.5)	4 (9.3)	0.434
Gallbladder carcinoma	5 (11.4)	0 (0)	0.055
Others	2 (4.5)	4 (9.3)	0.434
Blood examination			
Platelet count, ×10^3^/μL	17.6 (7.9–54.3)	19.3 (9.9–43.4)	0.271
Albumin, g/dL	4.1 (3.3–5.3)	4.3 (3.3–4.7)	0.112
Total bilirubin, mg/dL	0.8 (0.3–2.0)	0.7 (0.3–2.6)	0.363
Prothrombin activity, %	99 (36–120)	93 (11–113)	0.024
ALT, IU/L	20 (6–272)	20 (10–103)	0.589
Creatinine, mg/dL	0.86 (0.48–1.32)	0.81 (0.45–1.35)	0.647
eGFR	65 (36–108)	70 (42–113)	0.682
Liver functional reserve and fibrosis marker			
ICG‐R15, %	13.4 (1.6–56.9)	13.0 (2.1–91.8)	0.766
M2BPGi, COI	0.89 (0.14–4.00)	0.91 (0.24–3.32)	0.744
Maximum diameter of tumor, cm	3.5 (1.0–10.0)	2.6 (1.0–6.0)	0.160
Location for segmentectomy of the liver			
S1	1 (2.3)	0 (0)	
S2	0 (0)	4 (9.3)	
S3	0 (0)	10 (23.3)	
S4a	1 (2.3)	1 (2.3)	
S4b	1 (2.3)	1 (2.3)	
S5	11 (25.0)	9 (20.9)	
S6	5 (11.4)	5 (11.6)	
S7	2 (4.6)	6 (14.0)	
S8	23 (52.3)	7 (16.3)	
PS segment (S4a, S7, S8)	26 (59.1)	14 (32.6)	0.022

Abbreviations: ALT, alanine aminotransferase; BMI, body mass index; eGFR, estimated glomerular filtration rate; ICG, indocyanine green; ICG‐R15, indocyanine green retention rate at 15 min.; LS, laparoscopic segmentectomy; M2BPGi, Mac‐2 binding protein glycosylated isomers; OS, open segmentectomy; PS, postero‐superior; S, segment of the liver.

The study protocol was approved by the Institutional Review Board of the Gunma University Hospital (Approval number: HS2021‐193). The study was conducted in accordance with the principles of the Declaration of Helsinki. As this was a retrospective cohort study, the requirement for informed consent was waived and the study was opt‐out.

### 2.2 Surgical procedure of LS and methods of ICG fluorescent navigation

**FIGURE 1:**
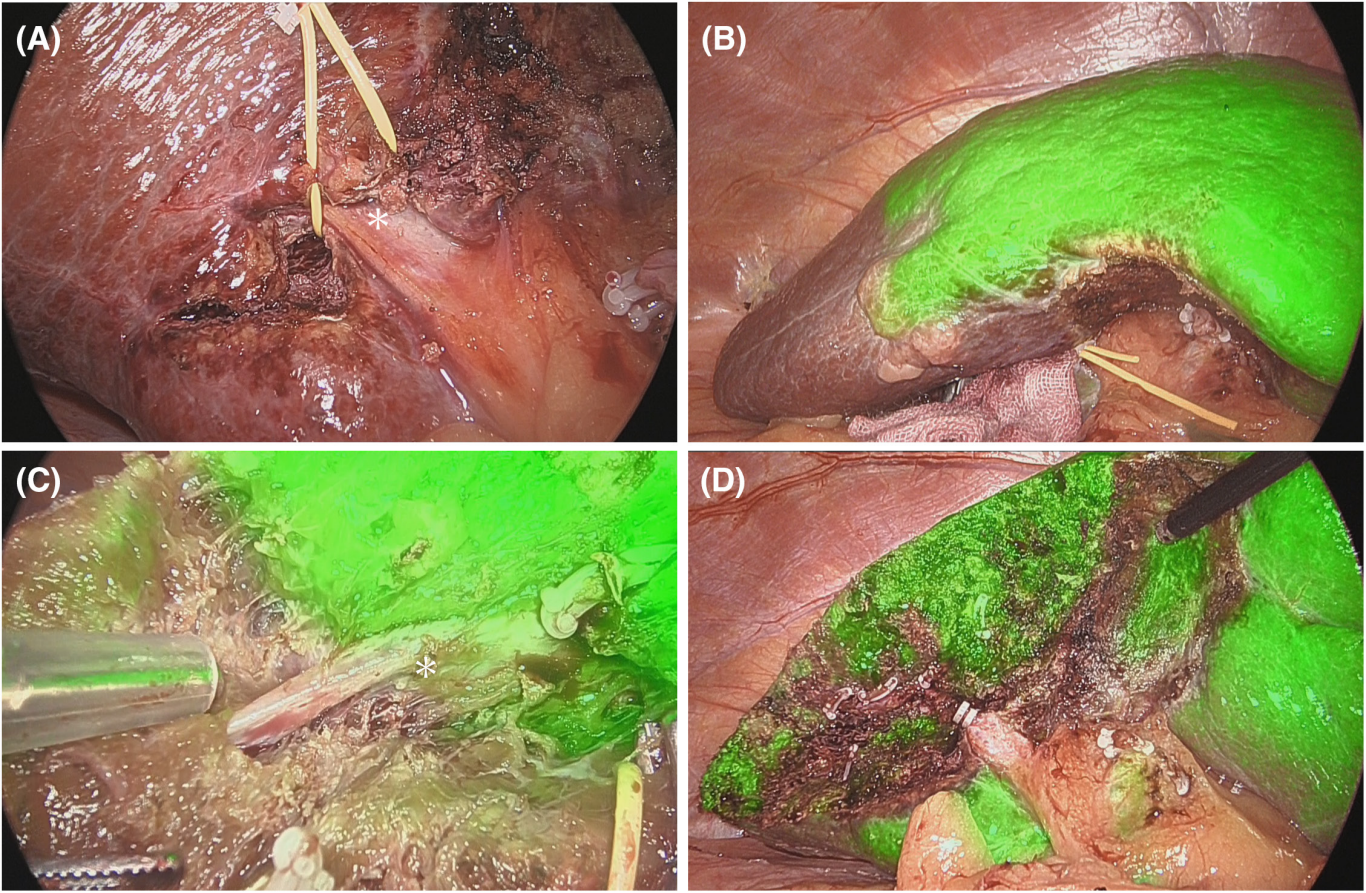
Laparoscopic segmentectomy (LS) of S5 using the ICG negative staining method. (A) Extrahepatic approach for Glissonean pedicle of S5. (B) Demarcation line was visualized using ICG fluorescence. (C) Liver parenchymal transection along the middle hepatic vein. The middle hepatic vein (*) is exposed from the cranial (root) to the peripheral parts. (D) Cutting surface after LS of S5. The stump of the G5 branch was exposed and the ICG fluorescent area was fully preserved.

**FIGURE 2:**
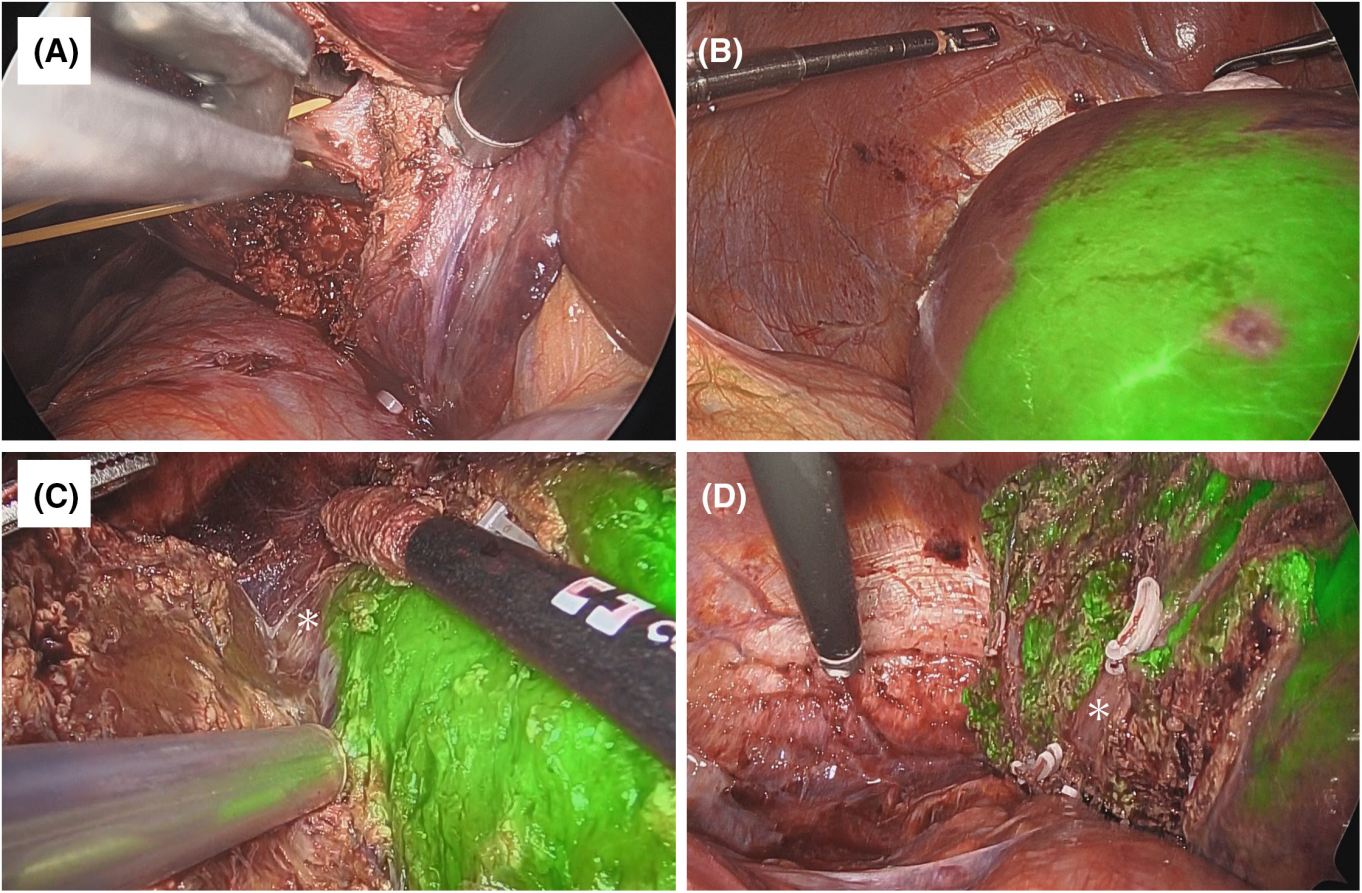
Images of laparoscopic segmentectomy (LS) of S7 using ICG negative staining method. (A) LS of S7 by intrahepatic Glissonean approach. Liver parenchymal transection from dorsal part of the liver. The Glissonean pedicle of S7 was exposed in a shallow layer. (B) Demarcation line is visualized by ICG fluorescence. (C) Visualization of intersegmental plane with inserting ICG rigid scope (45°) via subcostal port and CUSA via intercostal port. The root of right hepatic vein (*) and V7 branch are exposed along intersegmental plane. (D) The cutting surface after LS of S7. The right hepatic vein (*) and stump of V7 branch were exposed.

The general procedure for LLR has been described previously.^13^ The details of the LS procedure are as follows: patients were placed in the supine (segments S2, S3, and S4) or semi‐left lateral decubitus positions (segments S5, S6, S7, and S8). Typically, six or seven ports, including the port for Pringle taping, were used (Figure S1). In the case of the PS segment, the intercostal port via the 7th or 8th intercostal part and high‐angle (45 degree) laparoscope (Stryker Corp., Japan) are used when the surgical field of view is poor or the transection device is not well angled.^7^, ^13^, ^14^ Pneumoperitoneum pressure was maintained at 10 mmHg. Laparoscopic ultrasonography was systematically used during surgery.^15^ For anterolateral (AL) segments, such as S2, S3, S4b, S5, and S6, the Glissonean pedicle was approached using an extrahepatic approach (Figure 1). Briefly, for the Glissonean pedicle of S5, the G5 branch was initiated from the hilar portion, oriented by a small parenchymal transection, and reached the pedicle of S8. For the posterosuperior segments, such as S4a, S7, and S8, we approached the Glissonean pedicle using the intrahepatic approach (Figure 2). Briefly, for the Glissonean pedicle of S7, liver transection was initiated from the dorsal side of the liver after mobilization of the right lobe and reached the pedicle of S7 under intraoperative ultrasonography (IOUS) guidance. This intrahepatic strategy of approaching the Glissonean sheath was used to prevent injury to the pedicles during isolation of deeper pedicles, such as S7 or S8.^16^, ^17^ To perform LS correctly, we noted complete removal of the territory of the third‐order portal branches of a Couinaud segment.^18^ To recognize the segmentation of the target Glissonean pedicle, preoperative 3D reconstruction was performed using SYNAPSE VINCENT software (Fujifilm Medical, Japan) to precisely define the segmental borders in each patient.^19^

ICG fluorescence navigation was performed using an IR system in all LS cases. After isolating and encircling the target Glissonean pedicle, it was clamped using bulldog forceps or divided after clipping. Then, ICG dye (1.25–2.5 mg, adjusted depending on the camera system) was injected intravenously, and observed demarcation line of the liver surface and intersegmental plane of the transecting surface by IR mode of the camera. IR visualization was performed using the 1688 AIM Platform (Stryker Corp., Japan) or VISERA ELITE II (Olympus Corp., Japan). When the IR mode of the laparoscope was switched, liver transection was performed along the boundary of the colors as visualized by ICG fluorescence.

Liver parenchymal transection was performed using ultrasonically activated scalpels and a Cavitron Ultrasonic Surgical Aspirator (CUSA; Integra Corp., Tokyo, Japan). Hemostasis was performed using dual coagulation devices, which used the tip of the CUSA connected to a VIO 300D generator (ERBE GmbH, Germany) and bipolar forceps held in the operator's left hand, both in the soft‐coagulation mode. Generally, an intermittent pedicle clamp (Pringle method: 15 min clamping and 5 min reperfusion) is used during parenchymal transection. Airway pressure was maintained between 15 and 16 cmH_2_O to reduce central venous pressure and volume perfusion.

### 2.3 Surgical procedure of OS

For OS, surgical procedure was described as follows. Laparotomy was mainly performed through a J‐shaped incision. The Glissonean pedicle was mainly approached using an extrahepatic approach. At the discretion of the surgeon, in cases of deep Glissonean pedicle such as G7/G8, liver transection was sometimes performed prior to isolate the pedicle using IOUS guide. The surface of the liver was transected along the demarcation line due to ischemic changes after Glissonean pedicle clamping, and liver transection was performed with reference to preoperative 3D simulation and intraoperative anatomical findings. Liver parenchymal transection and hemostasis were performed with same methods of LS as described before.

### 2.4 Evaluation of preoperative computed tomography (CT) volumetry and resected liver volume

Preoperative CT imaging was routinely performed in four phases (two arterial phases, the portal phase, and the equilibrium phase: liver protocol) after contrast enhancement. The PRLV was calculated using SYNAPSE VINCENT volume analyzer software (described previously). After excision, the actual resected liver volume (ARLV) was quickly measured in the operating room using a digital scale.

### 2.5 Statistical analyses

Continuous data are presented as mean (SD) and median (range) for normally and non‐normally distributed variables, respectively. Differences between the two cohorts were assessed using the Mann–Whitney U test and Fisher's exact test, as appropriate. Correlations between the predicted and actual resected liver volumes were assessed using Spearman's correlation coefficients, as these variables exhibited non‐normal distributions. All statistical assessments were performed using IBM SPSS Statistics software version 28 (IBM Corp., Chicago, IL, USA). A distribution chart of the correlation and a graph of the boxplot were created using this software. Statistical significance was set at *p* value <0.05.

## 3 RESULTS

### 3.1 A comparison of patient characteristics between the OS and LS groups

The patient characteristics of the two groups are shown in Table 1. There were no differences in patient factors, such as age (*p* = 0.460), sex (*p* = 0.559), BMI (*p* = 0.316), and BSA (*p* = 1.000). In the preoperative diagnosis, gallbladder carcinoma cases tended to be more frequent in the OS group (*p* = 0.055), which suggests that LS is not indicated for gallbladder carcinoma that requires anatomical resection in our department. Among the laboratory findings, only prothrombin activity was lower in the LS group (*p* = 0.024). There were no differences in liver function and fibrosis indicators, including ICG‐R15 (*p* = 0.766), M2BPGi (*p* = 0.744), and tumor diameter (*p* = 0.160), between the two groups. Regarding the location of the liver segments, there were more PS segments in the OS group (*p* = 0.017).

### 3.2 A comparison of surgical outcomes between the OS and LS groups

The surgical outcomes of the two groups are shown in Table 2. The operative time was shorter in LS group (*p* = 0.023), although the intermittent clamping (Pringle's) time was longer in the LS group (*p* = 0.005). Blood loss was lower (*p* < 0.001) and the length of hospital stay was shorter (*p* < 0.001) in the LS group. There were no conversion cases, including hand‐assisted laparoscopic surgery (HALS) or small‐incision laparotomy (hybrid procedure), in the LS group. Both of the postoperative complications greater than grade II (*p* = 0.007) and grade III (*p* = 0.007) according to the Clavien–Dindo classification occurred in the OS group rather than in the LS group. Intra‐abdominal abscesses tended to occur in the OS group (*p* = 0.055). There was no difference in the occurrence of bile leakage between the two groups (*p* = 0.360). Bile leakage occurred in one case in the LS group. In the present case, LS was performed for S3, and bile leakage developed in the late phase after discharge and was ameliorated by percutaneous drainage. No mortality was observed in the present study.

**TABLE 2:** Surgical outcomes between OS and LS using ICG navigation.

	OS (*n* = 44)	LS using ICG navigation (*n* = 43)	*p* Value
Operative time, min.	380 (189–584)	339 (190–546)	0.023
Blood loss, mL	220 (33–722)	55 (0–575)	<0.001
Intraoperative blood transfusion	1 (2.3)	0 (0)	1.000
Intermittent clamping time, min.	104 (45–200)	138 (53–330)	0.005
Conversion (including HALS)	–	0 (0)	–
Postoperative complication			
Ascites	1 (2.3)	0 (0)	1.000
Intraabdominal abscess	5 (11.4)	0 (0)	0.055
Bile leakage	4 (9.2)	1 (2.3)	0.360
Pneumonia	0 (0)	0 (0)	–
Posthepatectomy liver failure	0 (0)	0 (0)	–
Clavien–Dindo ≥grade II	12 (27.3)	2 (4.7)	0.007
Clavien–Dindo ≥grade III	10 (22.7)	1 (2.3)	0.007
Mortality	0 (0)	0 (0)	–
Length of hospital stay, days	13 (8–134)	8 (6–17)	<0.001
Laboratory data on POD1			
Prothrombin activity, %	71 (65–94)	76 (39–102)	0.165
Total bilirubin, mg/dL	1.2 (0.4–5.6)	1.0 (0.5–2.8)	0.144
AST, IU/L	537 (174–1169)	318 (58–839)	<0.001
ALT, IU/L	435 (147–1030)	282 (49–823)	<0.001
CRP, mg/dL	3.38 (0.62–8.23)	1.46 (0.42–6.27)	<0.001
**PS segment**	**(*n* = 26)**	**(*n* = 14)**	
Prothrombin activity, %	64 (45–85)	76 (57–90)	0.051
Total bilirubin, mg/dL	1.3 (0.7–2.9)	1.2 (0.6–2.2)	0.332
AST, IU/L	553 (174–920)	366 (195–633)	<0.001
ALT, IU/L	484 (147–1030)	352 (171–636)	0.110
CRP, mg/dL	3.19 (0.62–8.23)	0.92 (0.42–6.27)	<0.001
**AL segment**	**(*n* = 18)**	**(*n* = 29)**	
Prothrombin activity, %	75 (50–94)	75 (39–102)	0.751
Total bilirubin, mg/dL	1.0 (0.4–5.6)	1.0 (0.5–2.8)	0.921
AST, IU/L	476 (188–1169)	280 (58–839)	0.005
ALT, IU/L	341 (153–895)	241 (49–823)	0.020
CRP, mg/dL	3.77 (1.20–7.73)	1.61 (0.44–6.27)	<0.001

Abbreviations: ALT, alanine aminotransferase; AST, aspartate aminotransferase; CRP, C‐reactive protein; HALS, hand‐assisted laparoscopic surgery; ICG, indocyanine green; LS, laparoscopic segmentectomy; OS, open segmentectomy; POD, postoperative day.

We also compared the postoperative laboratory findings between the two groups on postoperative day (POD) 1 (Table 2). There was no significant difference between the two groups in total bilirubin level (*p* = 0.144) or prothrombin activity (*p* = 0.165) on the first day after surgery. Aspartate aminotransferase (AST) (*p* < 0.001), alanine transaminase (ALT) (*p* < 0.001), and C‐reactive protein (CRP) (*p* < 0.001) levels were significantly lower in the LS group than in the OS group.

### 3.3 Evaluation of accuracy in resected liver volume between OS and LS using ICG navigation

**FIGURE 3:**
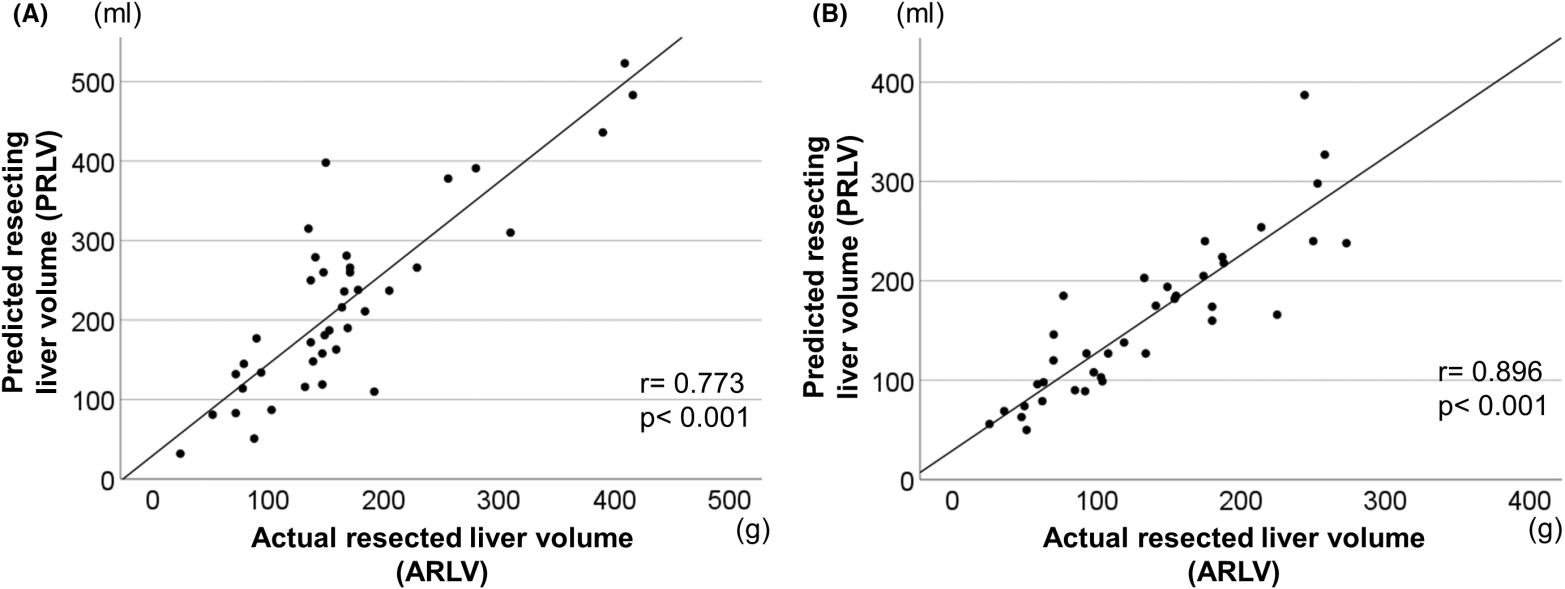
Correlation scatter plots between predicted resecting liver volume and resected liver volume in each group. The straight line indicates regression line. (A) Correlation in the open segmentectomy (OS) group. (B) Correlation in the laparoscopic segmentectomy (LS) using ICG navigation group. “*r*” indicates Spearman's correlation coefficient.

We also examined the correlation between the PRLV and ARLV in both groups. The correlation coefficient (*r* = 0.896, 95% confidence interval (CI): 0.804–0.946, *p* < 0.001) of LS using ICG navigation was superior to that of OS (*r* = 0.773, 95% CI: 0.604–0.875, *p* < 0.001) (Figure 3). Table 3 shows the results for the PRLV and ARLV, the difference between the PRLV and ARLV, and the error ratio calculated using the following formula: (PRLV‐ARLV)/PRLV×100. According to an analysis of all cohorts, PRLV (185 [32–443] ml vs. 138 [48–330] ml, *p* = 0.085) and ARLV (150 [24–490] ml vs. 114 [26–273] ml, *p* = 0.062) tended to be larger in the OS group, but not statistically significant. The difference between PRLV and ARLV was significantly smaller in the LS group (−38 [−248–82] vs. −30 [−108–59], *p* = 0.022). The error ratio did not differ significantly between the two groups (−22 [−62–75] vs. −15 [−58–36], *p* = 0.564) (Table 3).

**TABLE 3:** Relationship between predicted resecting liver volume and actual resected liver volume.

	OS	LS using ICG navigation	*p* Value
**All cohort**	**(*n* = 44)**	**(*n* = 43)**	
PRLV, mL	185 (32–443)	138 (48–330)	0.085
ARLV, g	150 (24–490)	114 (26–273)	0.062
Difference: ARLV ‐ PRLV	−38 (−248 to 82)	−30 (−108 to 59)	0.022
Error ratio^a^ (%)	−22 (−62 to 75)	−15 (−58 to 36)	0.564
**PS segment**	**(*n* = 26)**	**(*n* = 14)**	
PRLV, mL	196 (50–443)	166 (63–327)	0.435
ARLV, g	149 (72–416)	180 (48–273)	0.568
Difference: ARLV − PRLV	−52 (−180 to 37)	−15 (−70 to 59)	0.008
Error ratio^a^ (%)	−19 (−57 to 73)	−14 (−35 to 36)	0.024
**AL segment**	**(*n* = 18)**	**(*n* = 29)**	
PRLV, mL	146 (32–388)	120 (48–330)	0.309
ARLV, g	152 (24–490)	96 (26–253)	0.045
Difference: ARLV − PRLV	−35 (−248 to 82)	−32 (−108 to 7)	0.811
Error ratio^a^ (%)	−16 (−62 to 75)	−18 (−58 to 6)	0.305

Abbreviations: AL, antero‐lateral; ARLV, actual resected liver volume; ICG, indocyanine green; LS, laparoscopic segmentectomy; OS, open segmentectomy; PRLV, predicted resecting liver volume; PS, postero‐superior.

^a^ Error ratio (%) = (PRLV − ARLV)/PRLV × 100.

### 3.4 Assessment of PS segment between OS and LS using ICG navigation

We compared the surgical outcomes in the PS segment between the two groups (Table 4). Blood loss was lower (*p* = 0.003) and the length of hospital stay was shorter (*p* < 0.001) in the LS group. Postoperative complications greater than grade II occurred frequently in the OS group (*p* = 0.058). There were no cases of intra‐abdominal abscess (*p* = 0.263) or biliary leakage (*p* = 0.263) in the LS group, but the difference was not significant compared with that in the OS group.

**TABLE 4:** Characteristics and surgical outcome in patients of PS segment.

	OS (*n* = 26)	LS using ICG navigation (*n* = 14)	*p* Value
Variables			
Age, years	69 ± 10	74 ± 8	0.205
Sex male	22 (84.6)	11 (78.6)	0.471
BMI	24.0 ± 3.1	24.6 ± 3.1	0.721
BSA, m^2^	1.66 ± 0.16	1.64 ± 0.18	0.613
Diagnosis			
Hepatocellular carcinoma	17 (65.4)	5 (35.7)	0.101
Colorectal liver metastases	6 (23.1)	7 (50.0)	0.168
Intrahepatic cholangiocarcinoma	2 (7.7)	1 (7.1)	0.679
Gallbladder carcinoma	0 (0)	0 (0)	–
Others	1 (3.8)	1 (7.1)	0.623
Blood examination			
Platelet count, ×10^3^/μL	17.6 (7.9–34.2)	19.7 (9.9–31.0)	0.271
Albumin, g/dL	4.1 (3.5–5.3)	4.2 (3.3–4.6)	0.987
Total bilirubin, mg/dL	0.9 (0.4–2.0)	0.7 (0.5–2.6)	0.223
Prothrombin activity, %	98 (36–120)	92 (71–107)	0.626
ALT, IU/L	24 (12–272)	17 (10–38)	0.105
Creatinine, mg/dL	0.84 (0.48–1.32)	0.79 (0.49–1.35)	0.814
eGFR	65 (41–106)	68 (42–113)	0.888
Liver functional reserve and fibrosis marker			
ICG‐R15, %	14.9 (1.6–56.9)	15 (5.7–23.0)	0.814
M2BPGi, COI	1.03 (0.20–4.00)	1.03 (0.32–1.38)	0.592
Maximum diameter of tumor, cm	3.3 (1.0–9.7)	2.5 (1.3–5.9)	0.485
Surgical outcome			
Operative time, min	400 (272–552)	370 (201–451)	0.116
Blood loss, mL	228 (50–722)	71 (17–486)	0.003
Intraoperative blood transfusion	1 (2.3)	0 (0)	0.650
Intermittent clamping time, min.	120 (75–200)	144 (77–232)	0.162
Conversion (including HALS)	–	0 (0)	–
Postoperative complication			
Ascites	1 (2.3)	0 (0)	0.650
Intraabdominal abscess	3 (11.5)	0 (0)	0.263
Bile leakage	3 (11.5)	0 (0)	0.263
Pneumonia	0 (0)	0 (0)	–
Posthepatectomy liver failure	0 (0)	0 (0)	–
Clavien–Dindo ≥grade II	9 (34.6)	1 (7.1)	0.058
Clavien–Dindo ≥grade III	5 (19.2)	0 (0)	0.143
Mortality	0 (0)	0 (0)	–
Length of hospital stay, days	13 (9–134)	8 (7–14)	<0.001

Abbreviations: ALT, alanine aminotransferase; BMI, body mass index; eGFR, estimated glomerular filtration rate; HALS, hand‐assisted laparoscopic surgery; ICG, indocyanine green; ICG‐R15, indocyanine green retention rate at 15 min; LS, laparoscopic segmentectomy; M2BPGi, Mac‐2 binding protein glycosylated isomers; OS, open segmentectomy; PS, postero‐superior.

We examined the correlation between PRLV and ARLV in patients with PS segments in the two groups. Values for both the difference between PRLV and ARLV (OS: −52 [−180–37] vs. LS: −15 [−70–59], *p* = 0.008) and the error ratio (OS: −19 [−57–73] vs. LS: −14 [−35–36], *p* = 0.024) in the LS group were significantly smaller than those in the OS group (Table 3). We also examined the correlation between PRLV and ARLV in patients with AL segments in the two groups. Values for the difference between these two variables (OS: −35 [−248–82] vs. LS: −32 [−108–7], *p* = 0.811) and the error ratio (OS: −16 [−62–75] vs. LS: −18 [−58–6], *p* = 0.305) in the LS group were not different from those in the OS group (Table 3).

## 4 DISCUSSION

Our results suggest that LS using ICG navigation may provide a more precise resection than conventional OS in anatomical segmentectomy of the liver. This result was more pronounced in the PS segment. Negative ICG fluorescent staining clearly delineates the segmental boundaries and is useful for an almost complete anatomical excision of each segment. This effectiveness was remarkable in the system with a function of full‐color rendering called “overlay mode.” Considering that the remnant ischemic area after hepatectomy has been reported to adversely affect early recurrence and poor prognosis after resection of hepatocellular carcinoma,^20^ complete excision of the portal territories during anatomical resection is important in terms of oncological aspects. With the spread of LLR, it is possible that LS using ICG fluorescence navigation will occupy an even more important position. To the best of our knowledge, this is the first comparative study of LS with ICG navigation and conventional OS.

In addition, by using the Glissonean approach separately for intrahepatic and extrahepatic methods, damage to the Glissonean sheath was prevented and acceptable postoperative outcomes were obtained. In the LS group, it is possible that the frequency of postoperative complications was low due to the magnifying effect, securement of the Glissonean sheath with an emphasis on safety, and accurate excision based on the territories of the portal vein. If proficient, liver parenchymal transection is possible in the IR mode most of the time during parenchymal transection. Normal white light should be used for manipulation of the perihilar plate because small bile leakage from the caudate branch of the bile duct is not visible in IR mode. However, the normal white‐light mode during bleeding facilitates the identification of the bleeding point for safe hemostasis.

Interestingly, in this study, postoperative AST, ALT, and CRP levels were significantly lower in the LS group. These results were favorable, which is in contrast to the significantly longer Pringle time in the LS group than in the OS group. The low transaminase levels may be attributed to the fact that the liver parenchyma was transected at a precise intersegmental plane and no ischemic area remained in the LS using ICG navigation. Correlation analysis showed that ARLV was smaller than PRVL in the OS group, suggesting that the increase in these values in postoperative laboratory findings reflects the existence of a residual ischemic area in the OS group.

Previous case series studies on LS alone are scarce.^7^, ^21^, ^22^ Several methods have been proposed to approach the Glissonean pedicles, and it seems that there is a tendency to be roughly divided into “extrahepatic approach,” which preserves the Laennec's capsule^23^ and similar methods, or “intrahepatic approach” and “transfissural approach,” which precede liver parenchymal transection. Funamizu et al. reported that the accuracy of LS using ICG navigation was assessed, and the outcomes were similar for training and senior surgeons.^22^ Looking at the results of these reports, the incidence of bile leakage varies from 0% to 6.7%.^7^, ^21^, ^22^ The LS obtained using our method can be said to have relatively good results. At this point, it seems difficult to judge which Glissonean pedicle approach is better, and we believe that it is important for LS to be used properly, depending on the operating surgeon and the situation.

The results of this study show that the difference in accuracy between PRLV and ARLV was more pronounced in the PS segment than in the AL segment. Although all boundaries between liver segments are not flat, ICG fluorescence imaging is particularly effective in detecting boundaries that are difficult to recognize. While performing this new surgical procedure, we sometimes notice that the true intersegmental plane is not flat but rather different from our conventional concept. Segmental boundaries are particularly difficult to recognize due to the location of anatomical characteristics. In the anterior section, the dorsal lesion is more developed than the ventral lesion and its dorsal boundary appears to protrude on the right dorsal side.^24^ In addition, the right lobe of the liver is depressed in the retroperitoneal direction, that is, in the dorsal direction; therefore, the boundary between S7 and S8 is not a straight line but is depressed dorsally. Previous study investigated the intersegmental plane by 3D simulation reported that S8 tended to protrude 39° (median) in the lateral direction and the right subphrenic region of the liver was usually occupied by S8 but not S7.^25^ Based on these anatomical characteristics, the accuracy of the resected liver volume in the PS segment was possibly better in the LS group using ICG navigation as far as our results see. ICG navigation can accurately recognize these difficult‐to‐recognize boundaries in real‐time, and it is expected that an accurate resected liver volume can be obtained per preoperative 3D simulation for all segments.

The IOUS guide is very useful when transecting the liver while exposing the hepatic vein and when injecting color dye into the target portal vein,^5^ and these methods remain important techniques in the field of liver surgery. However, in laparoscopic surgery, there is no reason to not use ICG navigation, which allows visualization of the intersegmental plane because the ischemic area is shared on the same screen. Previously, experienced liver surgeons could predict the intersegmental plane based on the path of small Glissonean branches that appears on transecting surface, but with this method using overlay mode of ICG camera, it has a possibility that less experienced surgeons can accurately determine the intersegmental plane as real‐time navigation. Furthermore, the advantages of ICG fluorescence navigation will be further accelerated in terms of the caudal view and parenchymal transection from the central (hilar) to the peripheral sides, which are obvious advantages of LLR.

This study had a few limitations. First, this was a retrospective study conducted at a single institution and the number of cases was relatively small. In the future, a multicenter prospective study will be necessary. Second, there was selection bias due to the time of surgery. We introduced LS for anatomical segmentectomy in 2020, and OS gradually replaced LS. This may be the reason why the postoperative results were better in the LS group, but we believe that there was little bias because the difference was within a few years and all procedures were performed by the same surgical team in our department over the entire period. Third, the OS group may not be a good comparative control. Best practice should be compared to LS without ICG fluorescent navigation. Since ICG navigation was used from the time LS was introduced, we had no choice but to compare it with the OS group. This should be considered in future multicenter studies.

In conclusion, LS using ICG fluorescent navigation enabled the accurate recognition of segmental boundaries and accurate excision of each segment compared to conventional OS. LS may contribute to safer short‐term postoperative outcomes than OS.

## FUNDING INFORMATION

This research did not receive any specifc grant from funding agencies in the public, commercial, or not‐for‐proft sectors.

## CONFLICT OF INTEREST STATEMENT

Ken Shirabe, who is a co‐author of this article, is an editorial member in the *Annals of Gastroenterological Surgery*. The authors declare no other conflicts of interest for this article.

## ETHICS STATEMENT

Approval of the research protocol: The study protocol was approved by the Institutional Review Board of Gunma University Hospital (Approval number: HS2021–193).

Informed Consent: This study was conducted in accordance with the principles of the Declaration of Helsinki. As this was a retrospective cohort study, the requirement for informed consent was waived and was in the form of an opt‐out.

Registry and the Registration No. of the study/Trial: N/A.

Animal Studies: N/A.

## Supporting information


Figure S1

## References

[1] Buell JF , Cherqui D , Geller DA , O'Rourke N , Iannitti D , Dagher I , et al. The international position on laparoscopic liver surgery: the Louisville Statement, 2008. Ann Surg. 2009;250(5):825–830. 19916210 10.1097/sla.0b013e3181b3b2d8

[2] Wakabayashi G , Cherqui D , Geller DA , Buell JF , Kaneko H , Han HS , et al. Recommendations for laparoscopic liver resection: a report from the second international consensus conference held in Morioka. Ann Surg. 2015;261(4):619–629. 25742461 10.1097/SLA.0000000000001184

[3] Hasegawa K , Kokudo N , Imamura H , Matsuyama Y , Aoki T , Minagawa M , et al. Prognostic impact of anatomic resection for hepatocellular carcinoma. Ann Surg. 2005;242(2):252–259. 16041216 10.1097/01.sla.0000171307.37401.db PMC1357731

[4] Moris D , Ronnekleiv‐Kelly S , Rahnemai‐Azar AA , Felekouras E , Dillhoff M , Schmidt C , et al. Parenchymal‐sparing versus anatomic liver resection for colorectal liver metastases: a systematic review. J Gastrointest Surg. 2017;21(6):1076–1085. 28364212 10.1007/s11605-017-3397-y

[5] Makuuchi M , Hasegawa H , Yamazaki S . Ultrasonically guided subsegmentectomy. Surg Gynecol Obstet. 1985;161(4):346–350. 2996162

[6] Castaing D , Garden OJ , Bismuth H . Segmental liver resection using ultrasound‐guided selective portal venous occlusion. Ann Surg. 1989;210(1):20–23. 2662923 10.1097/00000658-198907000-00003 PMC1357760

[7] Ishizawa T , Gumbs AA , Kokudo N , Gayet B . Laparoscopic segmentectomy of the liver: from segment I to VIII. Ann Surg. 2012;256(6):959–964. 22968066 10.1097/SLA.0b013e31825ffed3

[8] Kovid N , Han HS , Yoon YS , Cho JY . Advanced laparoscopic HPB surgery: experience in Seoul National University Bundang Hospital. Ann Gastroenterol Surg. 2020;4(3):224–228. 32490336 10.1002/ags3.12323 PMC7240149

[9] Ishizawa T , Zuker NB , Kokudo N , Gayet B . Positive and negative staining of hepatic segments by use of fluorescent imaging techniques during laparoscopic hepatectomy. Arch Surg. 2012;147(4):393–394. 22508790 10.1001/archsurg.2012.59

[10] Aoki T , Yasuda D , Shimizu Y , Odaira M , Niiya T , Kusano T , et al. Image‐guided liver mapping using fluorescence navigation system with indocyanine green for anatomical hepatic resection. World J Surg. 2008;32(8):1763–1767. 18543027 10.1007/s00268-008-9620-y

[11] Itoh S , Tomiyama T , Morinaga A , Kurihara T , Nagao Y , Toshima T , et al. Clinical effects of the use of the indocyanine green fluorescence imaging technique in laparoscopic partial liver resection. Ann Gastroenterol Surg. 2022;6(5):688–694. 36091307 10.1002/ags3.12563 PMC9444859

[12] Nomi T , Hokuto D , Yoshikawa T , Matsuo Y , Sho M . A novel navigation for laparoscopic anatomic liver resection using indocyanine green fluorescence. Ann Surg Oncol. 2018;25(13):3982. 30218249 10.1245/s10434-018-6768-z

[13] Araki K , Harimoto N , Ishii N , Tsukagoshi M , Igarashi T , Watanabe A , et al. Optimal indications for an intercostal port for the superior segments in laparoscopic partial liver resection. Asian J Endosc Surg. 2020;13(3):382–389. 31468734 10.1111/ases.12753

[14] Ogiso S , Conrad C , Araki K , Nomi T , Anil Z , Gayet B . Laparoscopic transabdominal with Transdiaphragmatic access improves resection of difficult Posterosuperior liver lesions. Ann Surg. 2015;262(2):358–365. 25848711 10.1097/SLA.0000000000001015

[15] Araki K , Conrad C , Ogiso S , Kuwano H , Gayet B . Intraoperative ultrasonography of laparoscopic hepatectomy: key technique for safe liver transection. J Am Coll Surg. 2014;218(2):e37–e41. 24315651 10.1016/j.jamcollsurg.2013.10.022

[16] Okuda Y , Honda G , Kobayashi S , Sakamoto K , Homma Y , Honjo M , et al. Intrahepatic Glissonean pedicle approach to segment 7 from the dorsal side during laparoscopic anatomic hepatectomy of the cranial part of the right liver. J Am Coll Surg. 2018;226(2):e1–e6. 29128388 10.1016/j.jamcollsurg.2017.10.018

[17] Ome Y , Honda G , Doi M , Muto J , Seyama Y . Laparoscopic anatomic liver resection of segment 8 using intrahepatic Glissonean approach. J Am Coll Surg. 2020;230(3):e13–e20. 31783094 10.1016/j.jamcollsurg.2019.11.008

[18] Wakabayashi G , Cherqui D , Geller DA , Abu Hilal M , Berardi G , Ciria R , et al. The Tokyo 2020 terminology of liver anatomy and resections: updates of the Brisbane 2000 system. J Hepatobiliary Pancreat Sci. 2022;29(1):6–15. 34866349 10.1002/jhbp.1091

[19] Ishii N , Harimoto N , Kogure K , Araki K , Hagiwara K , Tsukagoshi M , et al. Study on the portal ramification pattern of the right anterior sector of the liver and a unique medial branch (PV8c) of the right anterior portal vein. Ann Gastroenterol Surg. 2022;6(5):679–687. 36091302 10.1002/ags3.12561 PMC9444865

[20] Cho JY , Han HS , Choi Y , Yoon YS , Kim S , Choi JK , et al. Association of remnant liver ischemia with early recurrence and poor survival after liver resection in patients with hepatocellular carcinoma. JAMA Surg. 2017;152(4):386–392. 28052154 10.1001/jamasurg.2016.5040 PMC5470428

[21] Kim JH . Laparoscopic anatomical segmentectomy using the transfissural Glissonean approach. Langenbeck's Arch Surg. 2020;405(3):365–372. 32388715 10.1007/s00423-020-01889-w

[22] Funamizu N , Ozaki T , Mishima K , Igarashi K , Omura K , Takada Y , et al. Evaluation of accuracy of laparoscopic liver mono‐segmentectomy using the Glissonian approach with indocyanine green fluorescence negative staining by comparing estimated and actual resection volumes: a single‐center retrospective cohort study. J Hepatobiliary Pancreat Sci. 2021;28(12):1060–1068. 33638899 10.1002/jhbp.924

[23] Sugioka A , Kato Y , Tanahashi Y . Systematic extrahepatic Glissonean pedicle isolation for anatomical liver resection based on Laennec's capsule: proposal of a novel comprehensive surgical anatomy of the liver. J Hepatobiliary Pancreat Sci. 2017;24(1):17–23. 28156078 10.1002/jhbp.410 PMC5299460

[24] Kogure K , Kuwano H , Fujimaki N , Ishikawa H , Takada K . Reproposal for Hjortsjo's segmental anatomy on the anterior segment in human liver. Arch Surg. 2002;137(10):1118–1124. 12361415 10.1001/archsurg.137.10.1118

[25] Shindoh J , Mise Y , Satou S , Sugawara Y , Kokudo N . The intersegmental plane of the liver is not always flat – tricks for anatomical liver resection. Ann Surg. 2010;251(5):917–922. 20395853 10.1097/SLA.0b013e3181d773ae

