# Supplementary material for: Advantages of laparoscopic segmentectomy of the liver using ICG fluorescent navigation by the negative staining method: A comparison with open procedure
Source: Ann Gastroenterol Surg. 2024 Mar 7;8(4):691–700. doi: 10.1002/ags3.12786 (PMC11216779; doi:10.1002/ags3.12786)
Supplement: Supplementary file 1 — Figure S1 [file AGS3-8-691-s001.zip › AGS3_12786_Legend.docx]

**Supplemental Figure 1**

**a:** Port placement in the right lobe of the liver (S5, S6, S7, S8). “C” indicates a 12 mm port, which mainly used for the laparoscope. “P” indicates 5 mm port for insertion of tourniquet for Pringle maneuver. A 12-mm trocar equipped with a balloon stopper was placed in the 7^th^ or 8^th^ intercostal space, as appropriate. **b:** Port placement in the left liver lobe (S2, S3, and S4). “P” indicates 5 mm port for insertion of tourniquet for Pringle maneuver.
