# Supplementary figures and images for: Advantages of laparoscopic segmentectomy of the liver using ICG fluorescent navigation by the negative staining method: A comparison with open procedure
Source: Ann Gastroenterol Surg. 2024 Mar 7;8(4):691–700. doi: 10.1002/ags3.12786 (PMC11216779; doi:10.1002/ags3.12786)

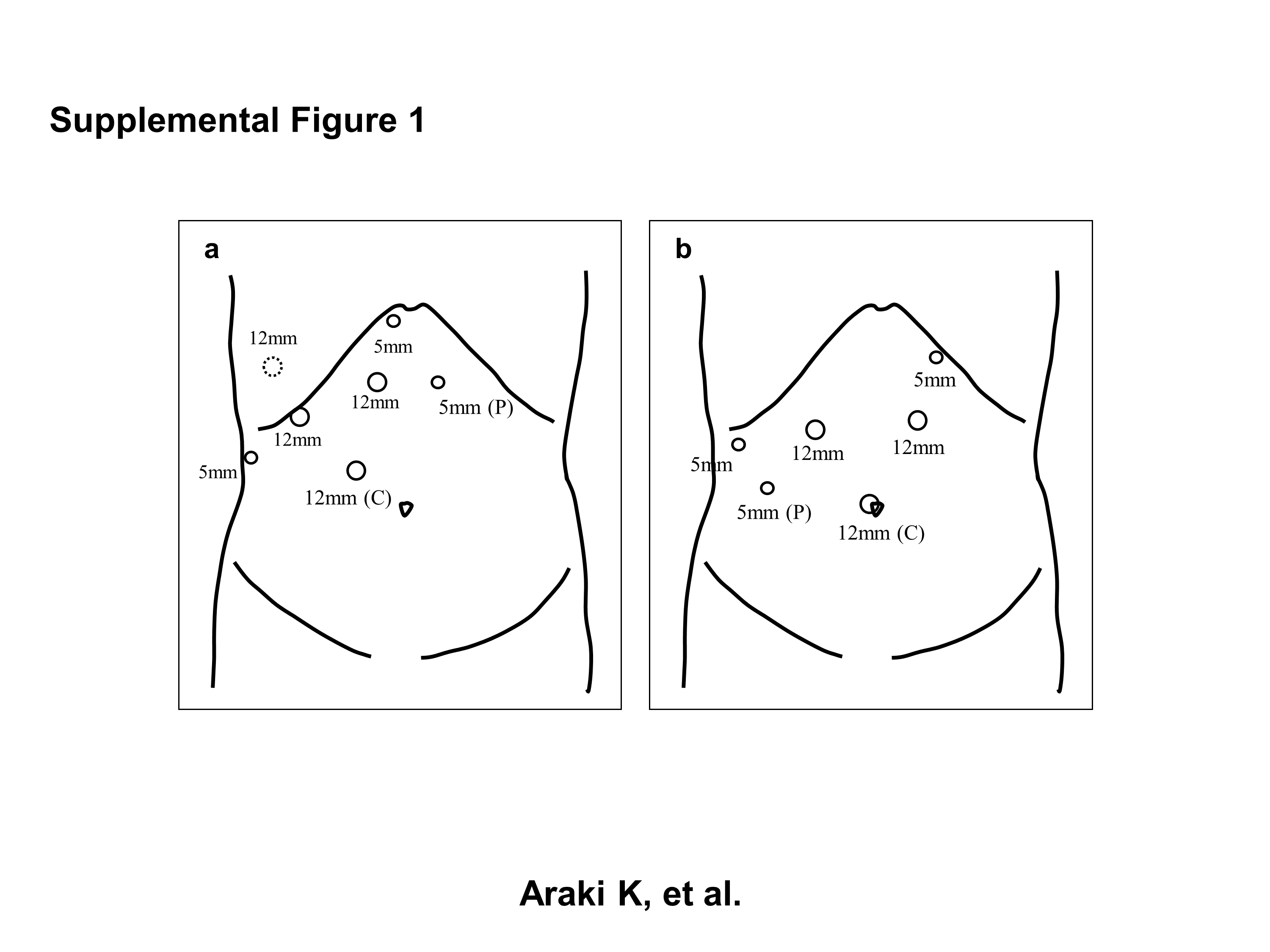

Supplement: Supplementary file 1 — Figure S1 [file AGS3-8-691-s001.zip › ags312786-sup-0001-FigureS1.TIF]
